# Screening for Dyslipidemia Among Patients Admitted With Acute Coronary Syndrome at the Jakaya Kikwete Cardiac Institute, Tanzania: A Retrospective Cohort Study

**DOI:** 10.7759/cureus.83200

**Published:** 2025-04-29

**Authors:** Naki Kiroga, Zahid Khan

**Affiliations:** 1 Department of Cardiology, Jakaya Kikwete Cardiac Institute, Dar es Salaam, TZA; 2 Department of Cardiology, University of South Wales, Pontypridd, GBR; 3 Department of Cardiology, The University of Buckingham, London, GBR; 4 Department of Cardiology, Barts Heart Centre, London, GBR

**Keywords:** acute coronary syndrome, atherosclerotic cardiovascular disease (ascvd), european society of cardiology/european atherosclerosis society, improved reduction of outcomes: vytorin efficacy international trial (improve it), jakaya kikwete cardiac institute, justification for the use of statins in prevention: an intervention trial evaluating rosuvastatin, low-density lipoprotein cholesterol (ldl-c), non-st-segment elevation myocardial infarction (nstemi), pravastatin or atorvastatin evaluation and infection therapy-thrombolysis in myocardial infarction 22, proprotein convertase subtilisin/kexin type 9 (pcsk9) inhibitors

## Abstract

Introduction: Dyslipidemia remains a significant risk factor for atherosclerosis and the development of acute coronary syndrome (ACS). Consistent data have demonstrated challenging lipid control according to the European Society of Cardiology (ESC) guideline-directed target low-density lipoprotein cholesterol (LDL-C) level. The exact prevalence of dyslipidemia in Tanzania remains unclear, although it is known to be quite high, and higher in urban than in rural areas. This study aimed to evaluate the current practice of lipid assessment in patients admitted with ACS and compliance with national and international guidelines.

Methodology: This retrospective hospital-based cohort study aimed to determine the current practice of dyslipidemia screening in patients with ACS admitted to the Jakaya Kikwete Cardiac Institute (JKCI) and evaluate their adherence to national and international recommended guidelines, such as ESC and American Heart Association guidelines. All patients admitted to the JKCI from June 2023 to June 2024, aged 18 years or older, and presenting with ACS, were included in this study. Data were collected using a prestructured tool created with Google Forms (Google, Mountain View, CA). Data extraction was performed in MS Excel (Microsoft Corporation, Redmond, WA) and then transferred to R software (R Foundation for Statistical Computing, Vienna, Austria) for analysis. This information is summarized in tables, graphs, and frequencies.

Results: This study included 124 patients diagnosed with ACS admitted to the JKCI center. Of this cohort, 58 (47%) patients had their LDL-C levels checked within 48 hours of presentation. The follow-up of lipids after admission was documented in 10% of the patients. Only 9.5% achieved the guideline-recommended LDL-C goal. Most patients were initiated on and maintained on a high-intensity statin, with no data on the use of nonstatin therapy.

Conclusion: The findings of this study have mirrored many irregularities in the current practice of dyslipidemia screening among patients with ACS admitted to JKCI. This study also highlighted the local nonadherence to national and international recommended guidelines, which, in turn, undermines secondary prevention of cardiovascular events. Further larger multicentered studies are recommended to assess the scope of the problem to reduce the burden and risk of future events.

## Introduction

Coronary artery disease (CAD) secondary to atherosclerosis is the leading cause of morbidity and mortality globally [1,2]. Atherosclerosis is a fibroproliferative disorder that plays an important role in the pathogenesis of acute coronary syndrome (ACS). It triggers inflammatory and immunological reactions in medium- and large-sized arteries, eventually accumulating total cholesterol, low-density lipoprotein cholesterol (LDL-C), triglycerides, and low levels of high-density lipoprotein cholesterol (HDL-C) [3].

Dyslipidemia, denoted by derangements in the triglycerides, LDL-C, total cholesterol, and HDL-C, has prognostic significance in patients with ACS. Various randomized controlled trials and epidemiological studies have suggested that a high level of LDL-C is a prime risk factor that should be targeted to reduce cardiovascular disease (CVD) [4]. Dyslipidemia causes atherosclerosis and eventually forms a plaque that may dislodge and completely occlude the vessel, causing ST-segment myocardial infarction or partially occlude it to cause non-ST-segment elevation myocardial infarction (NSTEMI) [5].

According to a World Health Organization (WHO) report, approximately 17.9 million deaths attributed to CVD were reported in 2019, representing 32% of all global deaths. ACS and stroke account for 85% of all deaths. Low- and middle-income countries account for three-quarters of deaths due to ACS and stroke [1]. Furthermore, among the 17 million premature deaths (under the age of 70 years) due to noncommunicable diseases in 2019, 38% were attributed to CVDs [6]. The WHO has classified countries into four categories based on the level of risk, and most African countries fall into the very high-risk category. The European Society of Cardiology (ESC) recommended Systematic Coronary Risk Evaluation 2-Older Persons for fatal and nonfatal 10-year CVD risk as low-to-moderate risk, high risk, and very high risk in patients <50 years, <2.5%, 2.5%-7.5%, and ≥7.5%, respectively. The American Heart Association (AHA) guidelines for fatal and nonfatal atherosclerotic cardiovascular disease (ASCVD) 10-year risk are divided into high ≥20%, intermediate ≥7.5% to 20%, borderline 5% to <7.5%, and low <5% [7].

ACS may present as ST-segment elevation, NSTEMI, or unstable angina. Acute myocardial infarction (AMI) is classified into five distinct types, and the type is due to plaque rupture or erosion of a blood vessel, resulting in thromboembolism, leading to occlusion of the blood vessel supplying blood to the heart muscles. Dyslipidemia plays an important role in the formation of such plaques. New advances in the management of ACS with coronary angiography have led to improved outcomes in terms of mortality and morbidity [8]. Patients who present with ST-elevation myocardial infarction (STEMI) are younger than those with NSTEMI [9].

Patients who have previously succumbed to ACS are at risk of further acute cardiovascular events within two years, with a mortality rate of 19%-22% within five years. The risk of mortality increases with the recurrence of ACS [10,11]. Prevention and proper management of ACS have contributed to its decline over the past years [12,13]. Multiple studies have revealed a high prevalence of dyslipidemia in patients admitted with ACS [14-17].

The medical management of ACS has centered on the proper and timely management of LDL-C [18]. The 2019 ESC/European Atherosclerosis Society (ESC/EAS) guideline on the management of dyslipidemia has recommended a lipid reduction of equal to or more than 50% from baseline and LDL-C goal of less than 1.4 mmol/L in very high-risk patients such as those patients with ACS to reduce the risk for developing ASCVD [19]. The lipid profile should be obtained within 48 hours of admission, with subsequent follow-ups conducted at four to six weeks to monitor the trend of low-density lipoprotein levels and at three to six months to assess drug adherence to statins [10,20]. The 2018 AHA/American College of Cardiology (AHA/ACC) guidelines for the management of blood cholesterol also make similar recommendations [21].

The National Institute for Health and Care Excellence (NICE) guidelines recommend using lipid-lowering therapy (LLT) for both primary and secondary prevention, aiming primarily to lower HDL-C by 40% from baseline. The guideline has a target LDL-C level of 2 mmol/L or less and non-HDL-C of 2.6 mmol/L or less, with the initial screening 24 hours after admission for ACS and reassessment within two to three months for the response after high-intensity statin therapy [22]. The first EAS Lipid Registry of Africa observational study aimed to identify and report risk factors and outcomes of premature ACS in patients in Sub-Saharan Africa, further understanding the risk and prevalence of ACS to improve standardized healthcare practices across Africa. There is a lack of studies regarding dyslipidemia prevalence in the African continent due to a lack of resources [23].

Prevalence of dyslipidemia in patients admitted with ACS

According to WHO estimates, in 2008, the prevalence of dyslipidemia in the adult population was estimated to be highest in Europe (53.7%) and the Americas (47.7%) [24,25]. The global death count due to CVD has increased from 12.4 million in 1990 to 19.8 million in 2022, reflecting global population growth and aging, and dyslipidemia (LDL-C) was included among the 15 most common risk factors for CVD in the Global Burden of Disease 2023 report [26]. Meanwhile, South Asia (30.3%) and Africa (23.1%) had lower prevalence. The reports also demonstrated significant differences in prevalence among various Asian Pacific countries, ranging from 9% in Indonesia to 46.9% in the Philippines. The prevalence ranges of HDL-C, high triglycerides, and LDL-C were 7.8%-47.2%, 13.9%-38.6%, and 10.1%-71.3%, respectively [23]. Furthermore, a systematic review of dyslipidemia in Africa revealed that the prevalence of elevated LDL-C was 25.5%, elevated triglycerides was 17%, and HDL-C was 37.4% [24].

A study on the patterns and prevalence of dyslipidemia among patients with ACS showed that dyslipidemia was highly prevalent among patients with unstable angina (64.3%), followed by NSTEMI (48.1%) and STEMI (45.3%) [27].

In Pakistan, 83.5% of patients with diabetes have dyslipidemia. Similar findings were observed in a study in Nepal, with an 88.1% prevalence of dyslipidemia. A previous study reported that the prevalence of dyslipidemia was 48.6%, which was relatively low compared to our findings [27]. Similar results were observed in India, where at least 79% of dyslipidemia was observed [17]. These patients had comorbidities and other risk factors such as diabetes, hypertension, and smoking [28]. Among lipids, LDL-C was found to be comparatively high in patients with ACS [28].

A study in Africa showed suboptimal control of LDL-C levels in patients with ACS [29]. High lipid levels in the background of diabetes, obesity, and hypertension were found to be significant determinants of dyslipidemia [15]. Similar findings were reported in Italy, with more than 52.5% of patients having an LDL-C level above 70 mg/d [30]. In the United States, patients with coronary heart disease (CHD) and CHD equivalent, termed high-risk patients, had poor control of LDL-C of >100 mg/dL, contrary to the guideline-recommended target levels [31]. This study aimed to identify gaps in the current clinical practice by comparing it with national and international guidelines and identify areas for intervention to improve patient care and outcomes.

## Materials and methods

Problem statement

Molecular and cellular studies have established a central role of LDL-C in the pathogenesis of atherosclerotic plaques and their clinical sequelae, including CHD and ischemic stroke. Epidemiological data have confirmed an independent positive association between LDL-C and CVD risk, suggesting a strong association between high LDL-C levels and future CVD events [30]. Patients with ACS often present to the hospital with a clinical emergency requiring emergency coronary angioplasty and revascularization. The focus on attempting coronary angiography and possible percutaneous intervention tends to mask the immediate assessment of lipid profile within 48 hours and later, and to evaluate the response to statin therapy to attain the guideline-recommended target LDL-C levels, leading to poor cardiovascular outcomes. Dyslipidemia, mainly high LDL-C, has been associated with a higher risk of future cardiovascular events, and NICE guidelines recommend a reduction of at least 40% in the LDL-C level as part of secondary prevention. The ESC guidelines recommend an LDL-C level of <70 mg/dL (<1.8 mmol/L) for high-risk patients and <55 mg/dL (<1.4 mmol/L) for very high-risk patients, respectively, to minimize the risk of future cardiovascular events.

Rationale of the study

Lipid management plays a vital role in the pathogenesis and management of ACS, and data regarding this topic are scarce in the Sub-Saharan African continent. There is a lack of available data regarding the assessment and management of dyslipidemia in patients with CAD in Tanzania. Maintaining target guideline-recommended lipid levels, particularly LDL-C, is crucial for the management and secondary prevention of ACS. This retrospective study aimed to assess adherence to guideline-recommended screening and management of dyslipidemia in patients admitted with ACS at the Jakaya Kikwete Cardiac Institute (JKCI). This was in accordance with the 2019 ESC/European Atherosclerosis Society (EAS) Guidelines for the management of dyslipidemias: Lipid Modification to Reduce Cardiovascular Risk: The Task Force for the Management of Dyslipidemias of the ESC and EAS.

Primary objective

The primary objective of this study was to evaluate the current practice of initial and follow-up screening of patients for dyslipidemia admitted with ACS to JKCI and to compare adherence with the ESC recommended guidelines. This study also evaluated management adherence in ACS patients with the ESC guidelines.

Secondary objectives

The secondary objective was to determine the proportion of ACS patients who underwent guideline-recommended treatment for dyslipidemia during admission and the follow-up period. Additionally, this study also looked at LLT optimization in these patients during the follow-up period.

Methodology

This study included all patients admitted to the JKCI with ACS between June 2023 and June 2024. The study was conducted to assess dyslipidemia screening and management based on the recommendations for screening and management of Dyslipidemia in ACS patients, as outlined in the 2019 ESC/EAS guidelines for the management of dyslipidemias and the EAS guidelines. Data were collected for total cholesterol, LDL-C, HDL-C, and non-HDL cholesterol, along with data for other relevant comorbidities. Patients with missing data or incomplete data were excluded from the study to minimize the risk of bias.

Inclusion criteria

All patients admitted at the JKCI from June 2023 to June 2024, aged above 18 years, admitted with ACS, and irrespective of whether they had coronary angioplasty or previous lipid assessment, were included.

Exclusion criteria

Patients admitted with noncoronary syndrome, patients undergoing routine angiography, patients who did not undergo blood lipid tests, patients who had lipid profiles checked for noncoronary reasons, those with stable angina, and children under 18 years of age were excluded.

Data collection and analysis

Patients' data were extracted from the hospital's electronic and paper records. We created a Google Excel sheet (Microsoft Corporation, Redmond, WA) for data extraction that consisted of various variables, including patients' demographic data, presentation, lipid assessment, statin type and dosage, and follow-up lipid screening and dosage optimization. We also collected data about patients' other relevant comorbidities. The data obtained included patients' baseline characteristics of the population, such as hospital number, age, sex, employment, education status, insurance status, date of admission and discharge, length of inpatient hospital stay, ethnicity, lipid assessment, and familial history of hypercholesterolemia. Additionally, the dosage of statin prescribed, patients' diagnoses, relevant comorbidities, medication prescribed, baseline LDL-C levels within 48 hours after presentation, timing of lipid screening, follow-up lipid screening, and type of statin prescribed were also included. Further data regarding the lipid profile, LDL-C level, and initial management of dyslipidemia, as well as a plan of action for dyslipidemia, including follow-up plans and dosage optimization, were collected. Data were collected and transferred to an online Google Form (Google, Mountain View, CA) and analyzed by using R statistical software (R Foundation for Statistical Computing, Vienna, Austria) for comorbidities, proportion of patients who had initial lipid screening within 48 hours of admission, statin dosage, and follow-up lipid assessment and optimization of statin and other LLT. Patients with missing data were excluded from this study. The data were counter-checked by the second researcher for any missing data and accuracy, and the results were summarized and displayed as graphs, frequencies, and proportions. About eight patients had incomplete or missing data; hence, these patients were excluded from the study. Finally, data regarding patients' employment and educational level were not documented in medical records.

Ethical consideration

Permission to conduct this retrospective study was obtained from the ethics committee of the JJKCI and the University of Buckingham's ethics committee. The ethics committee approval number for this study was AB.123/307/01L/11.

## Results

Between June 2023 and June 2024, 132 patients were admitted with the diagnosis of ACS. Eight patients were excluded from the study due to missing data, with 124 patients included. The mean age of the study population was 61.50 years (mean age ± standard deviation, 67.5 ± 8.40), characterized by a predominance of male participants, totaling 100 (81%), in contrast to female participants, numbering 24 (19%). Patients covered by insurance were 69 (56%), while noninsured patients were 55 (44%). The majority of the patients, 109 (88%), presented with STEMI, while 10 (8.1%) had unstable angina, and five patients (4%) had an NSTEMI.

Results also display a lack of proper documentation on patients’ backgrounds that would have helped anticipate the causes of the diseases. Most patients’ records lacked information on their levels of education and employment status; 121 (98%) of the study population had no information about their educational background, and 78 (63%) had no information on employment status. The median number of days of admission was four days (interquartile range, 3-6 days). In all these admitted patients, the familial history of hypercholesterolemia was not documented in the admission notes of 122 (98%), as shown in Table 1.

**Table 1:** Baseline demographic and clinical characteristics of patients IQR: interquartile range; ACS: acute coronary syndrome; NSTEMI: non-ST-elevation myocardial infarction; STEMI: ST-segment elevation myocardial infarction

Characteristic	Total
Total patients	124
Age in years, median (IQR)	61.50 (51.75-68.25)
Sex, n (%)	
Female	24 (19%)
Male	100 (81%)
Employment, n (%)	
Agriculture	1 (0.8%)
Formal employment	3 (2.4%)
Not documented	78 (63%)
Retired	35 (28%)
Self-employment	7 (5.6%)
Education, n (%)	
Not documented	121 (98%)
Others	3 (2.4%)
Status of insurance, n (%)	
Insured	69 (56%)
Not insured	54 (44%)
Number of days of admission, median (IQR)	4 (3-6)
Familial history of hypercholesterolemia, n (%)	
No	2 (1.6%)
Not documented	122 (98%)
ACS type, n (%)	
NSTEMI	5 (4%)
STEMI	109 (88%)
Unstable angina	10 (8.1%)

**Figure 1:**
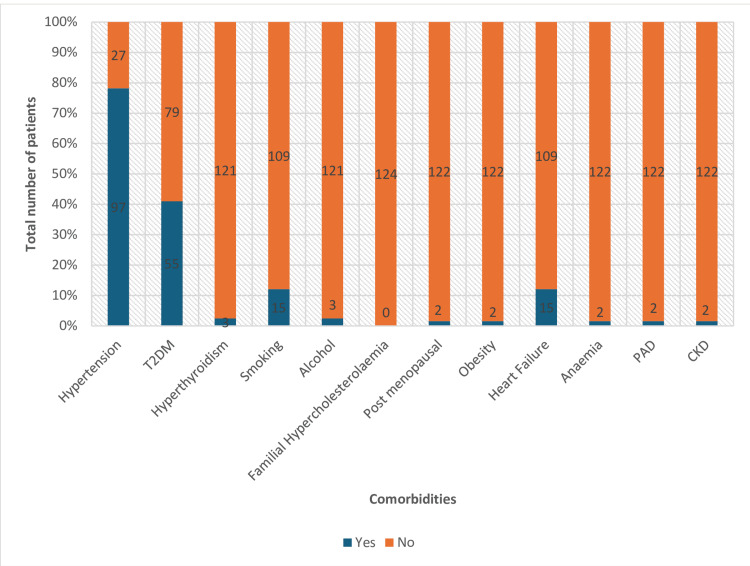
Patients' underlying comorbidities T2DM: type 2 diabetes mellitus; CKD: chronic kidney disease

It is observed that the most prevalent cardiovascular risk factors documented in the hospital system among the patients were hypertension in 97 (78%) and type 2 diabetes mellitus in 55 (45%). The other risk factors that were poorly documented include a history of smoking in 15 patients (12%), alcohol consumption in three patients (2.4%), obesity in one patient (0.8%), and postmenopausal status for women in one patient (0.8%). The history of familial hypercholesterolemia was not widely documented in most 122 (98%) patients. In this study, the comorbidities recorded among the patients were heart failure with reduced ejection fraction in 15 patients (12%), anemia in one patient (0.8%), peripheral arterial disease in one patient (0.8%), and chronic kidney disease stage 1 in one patient (0.8%) (Figure 1). About 45% of patients had multiple comorbidities, with hypertension, diabetes mellitus, and smoking the three most common conditions.

**Figure 2:**
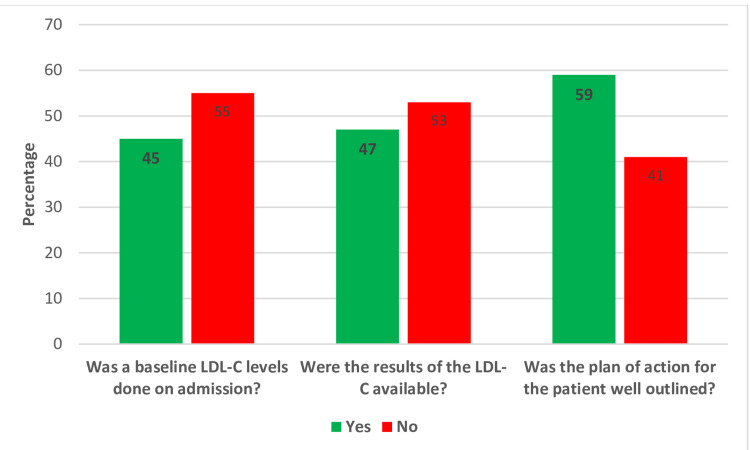
LDL-C baseline and follow-up assessment LDL-C: low-density lipoprotein cholesterol

Moreover, regarding the lipid profile, only 55 (45%) patients had their lipid profile assessed on admission, with a mean LDL of 2.83 mmol/L, while the lipid profiles of 48% of patients were not evaluated. Overall, 47% of patients underwent a lipid profile assessment within 48 hours after admission with ACS. The results of the lipid assessment were available and documented in the system for only 58 (47%) patients, and 59% of patients had their plan of action for lipid management well outlined. This accounts for <50% of the lipid assessment and management requirement per the ESC guidelines (Figure 2).

**Figure 3:**
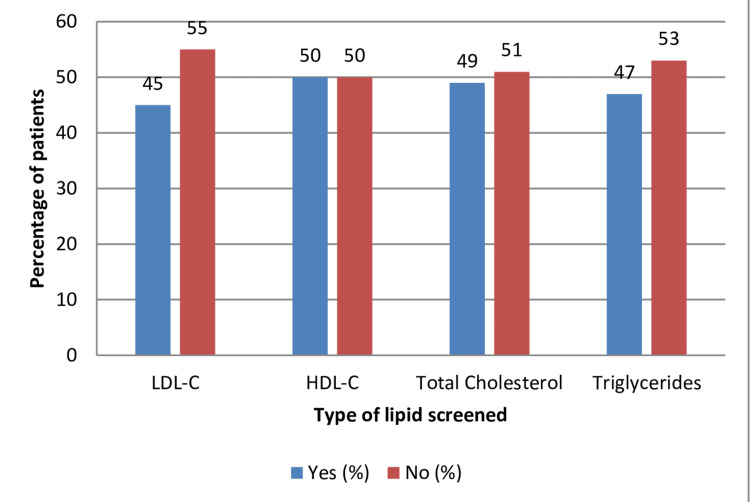
Percentage of patients that screened for all lipids on admission LDL-C: low-density lipoprotein cholesterol; HDL-C: low-density lipoprotein cholesterol

Patients were also screened for other types of lipids, including total cholesterol, HDL-C, and triglycerides. Among the 124 patients, 49% (61) were screened for total cholesterol, 50% (60) were screened for HDL-C, and 47%(59) were screened for triglycerides. Overall, nearly half of all patients had at least all types of lipids screened upon admission (Figure 3).

Most patients (94%) were initiated on high-intensity statin therapy after attending the institute. The statin was initiated on admission to 98% of patients. A total of 105 (85%) received atorvastatin 40 or 80 mg, while 19 (15%) received rosuvastatin 20 or 40 mg. All the patients had their statins given at the right frequency. About 113 (90.6%) admitted patients had no follow-up lipid profile done in three to six months, with subsequent lack of documentation of the follow-up lipid profile results in 105 (91%).

**Figure 4:**
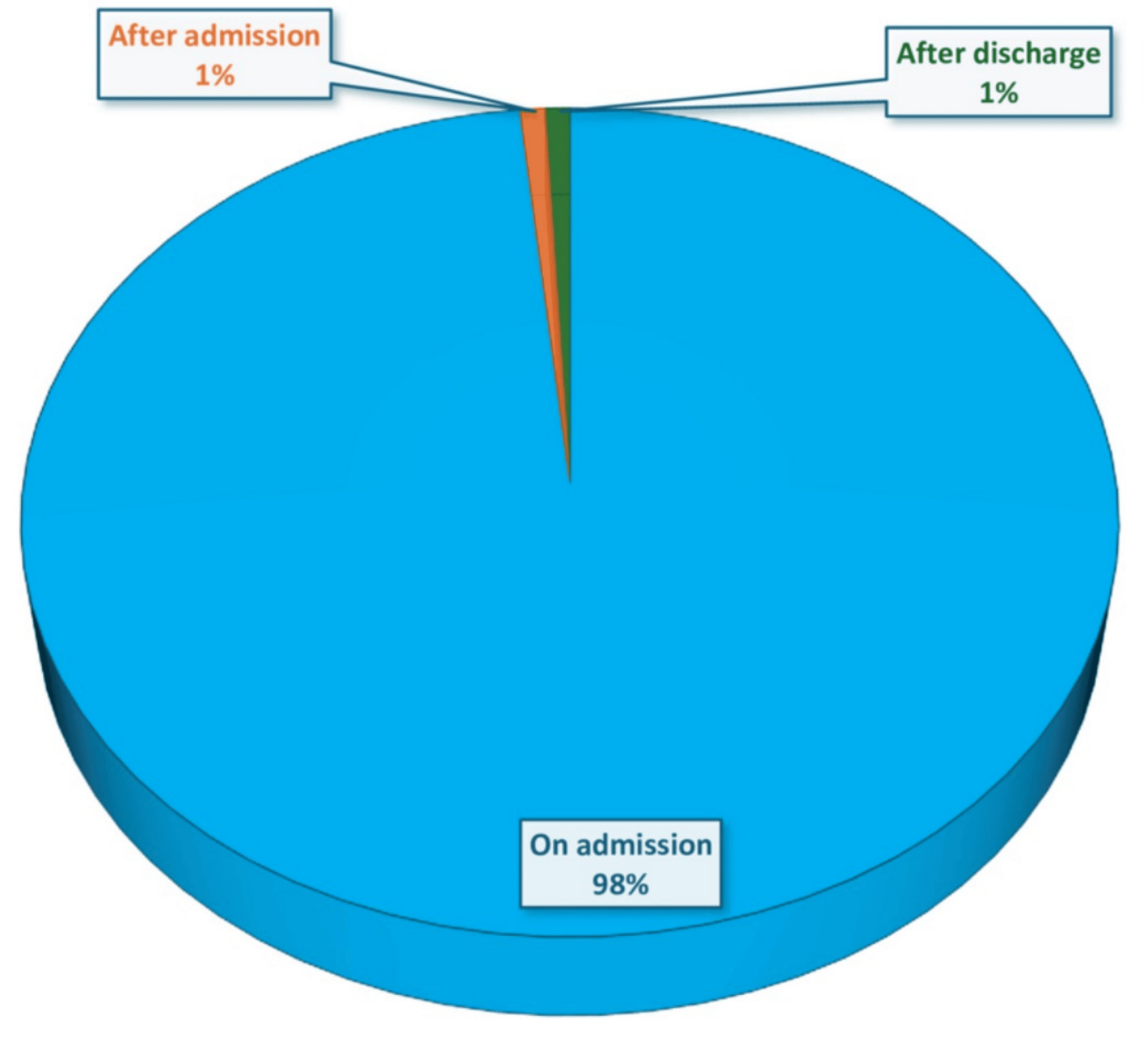
Proportion of patients showing the time of the prescription of statin in patients with acute coronary syndrome

**Figure 5:**
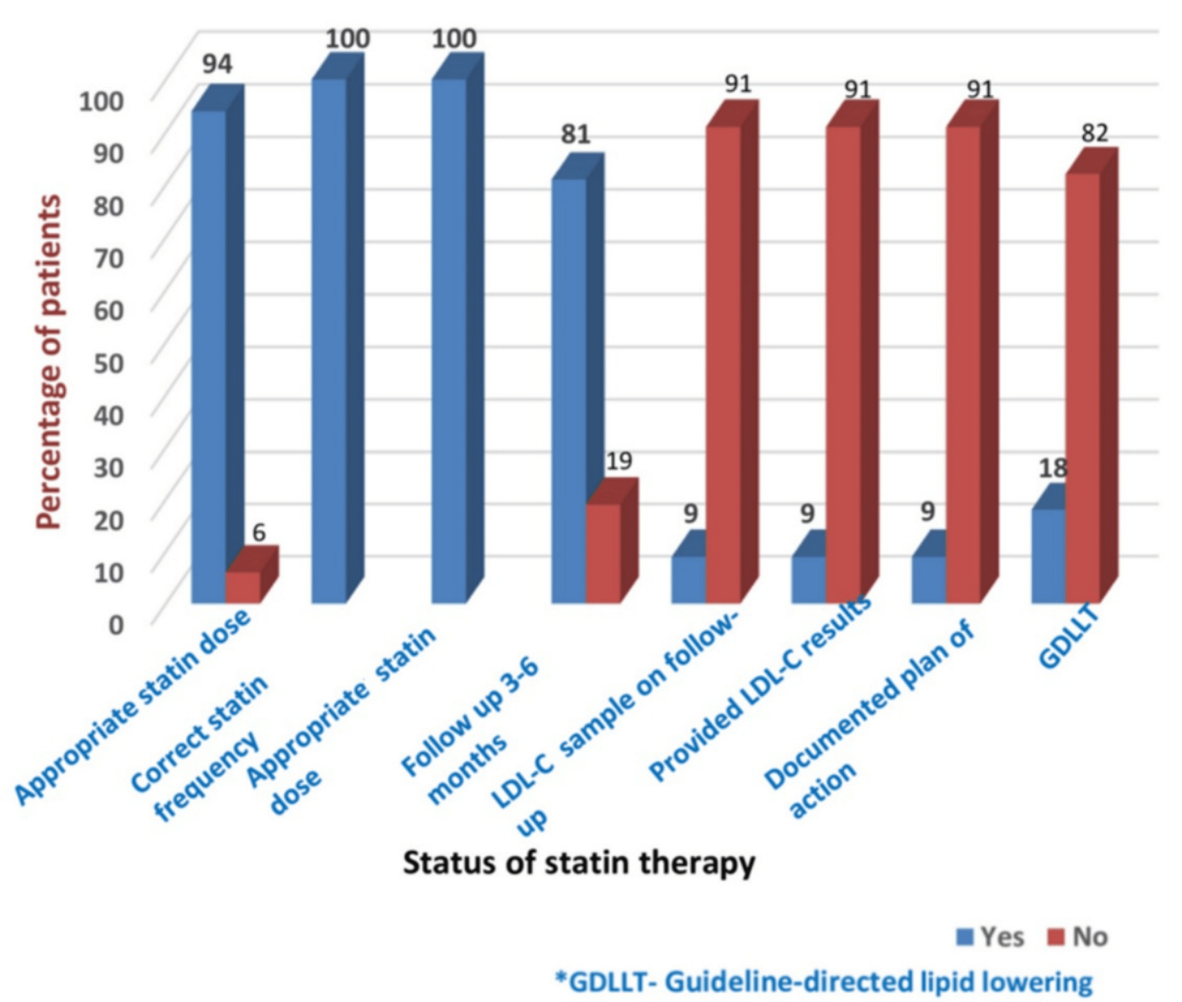
Pattern of statin therapy LDL-C: low-density lipoprotein cholesterol

Among the patients who underwent lipid profile analysis, only two (18%) patients achieved the guideline-recommended LDL-C goal of an LDL-C target of <1.4 mmol/L and a ≥50% reduction from baseline LDL-C. The remaining 82% had suboptimal LDL-C levels. Upon follow-up of statin therapy maintenance, 101 (81%) patients were on statin monotherapy. None of the patients was kept on any other nonstatin lipid-lowering medications (Figures 4, 5).

## Discussion

Different studies have been conducted worldwide to reflect actual practice and provide an objective way to standard practice to prevent cardiovascular events in patients. This is the first local study at the JKCI Hospital and in the country as a whole that has been done to assess adherence to the ESC guidelines for the management of dyslipidemia for primary or secondary prevention of ACS. The main findings of this study mirror some of the key messages that have tremendous implications regarding the existing situation and the adherence to the ESC guidelines. These include the following.

The study shows that most of the population admitted to JKCI Hospital had a diagnosis of STEMI, 109 (81%). The male patients’ predominance has been observed in comparison to female patients. These findings were similarly observed in other studies from developing countries, with the most prevalent risk factors that were documented in the study population being hypertension (48%) and type 2 diabetes (45%) [18]. In Pakistan, among patients with diabetes, 83.5% of the patients had dyslipidemia, while a study in Nepal showed 88.1% of dyslipidemia [26].

In this study, the majority of the patients had a diagnosis of STEMI, followed by NSTEMI, and, finally, unstable angina. While some studies revealed a similar picture, others had the patterns and prevalence of dyslipidemia among ACS patients that showed that dyslipidemia was highly prevalent among patients with unstable angina (63.4%), NSTEMI (48.1%), and STEMI (45.3%) [26,27]. A large body of evidence has demonstrated a clear relationship between the levels of LDL-C and the risk of ASCVD. Furthermore, the reduction in ASCVD risk is proportional to the absolute reduction in LDL-C [27-32].

The 2019 ESC/EAS guidelines for the management of dyslipidemias and the 2023 ESC Guideline for the management of ACS recommend a stepwise approach to LLT with a reevaluation of LDL-C goals after four to six weeks. A high-intensity statin therapy should be initiated in all ACS patients, regardless of initial LDL-C values, with a goal LDL-C of <55 mg/dL (<1.4 mmol/L) and a reduction of at least 50% from baseline [11].

If at all these goals are not met with high-intensity statin therapy alone, ezetimibe, and if goals are still not met, a proprotein convertase subtilisin/kexin type 9 (PCSK9) inhibitor should be added [33-35]. This study observed that almost half of the patients admitted had their LDL-C checked within 48 hours of presentation to the hospital. This shows considerable adherence to lipid screening at the time of admission. The guideline recommends that all patients with ACS have their LDL-C checked within 48 hours. The practice from this study unveils an awareness of guideline adherence, although there still seems to be a gap in the remaining half of the practitioners [35].

The data from this study revealed that most statin therapy patients were given appropriately, in terms of the correct dosage and frequency. The majority of patients were prescribed high-intensity statin therapy. Nonstatin therapy was underutilized due to the country's limited availability and high cost.

Evidence has already revealed that LDL-C serum concentrations are associated with cardiovascular risk. Intensive statin treatment is recommended to reduce the rate of recurrent ischemic events and stent thrombosis in patients with ACS [36]. Every 1.0 mmol/L reduction in LDL-C is associated with a corresponding 20-25% reduction in cardiovascular mortality and non-fatal myocardial infarction. According to the current ESC and ACC/AHA guidelines, a treatment goal of LDL-C < 70 mg/dL is recommended. Despite the emphasis of guidelines on the tight control of the LDL-C level, several surveys have shown that many patients remain undertreated and do not attain LDL-C treatment goals [37]. Similar results are displayed in this study.

The ESC guideline on managing dyslipidemia also recommends obtaining a lipid profile within 48 hours of admission, with subsequent follow-up at four to six weeks and three to six months for drug adherence. From this study, only 11 (9.4%) of all the patients recruited had a follow-up lipid profile in three to six months, with subsequent lack of documentation of the follow-up lipid profile results in 105 (91%) patients. With little or no follow-up for four to six weeks and three- to six-month follow-up of LDL-C levels after the initiation of statins, the study also shows there is little or no adherence to guidelines on patient follow-up after discharge. This is due to a lack of poor follow-up planning, limited resources, poor familiarity with guidelines, and poor documentation. Addressing these factors and incorporating them into the cardiac rehab program can perhaps improve patient care and compliance with guidelines.

Regarding documentation of the historical background of the patients, out of 124 patients, 121 (98%) in this study population were not documented to have an educational background. In contrast, 78 (63%) patients did not have information on their employment status. In the EmploYEd antithrombotic therapies in patients with acute coronary Syndromes HOspitalized in iTalian cardiac care units registry, a multivariate analysis revealed that a low level of education was associated with poor LDL-C control above 70 mg/mL [4]. The lack of this information, as observed in his study, hinders an opportunity to establish a correlation between the patients’ background and efforts to control LDL-C.

Moreover, the proportion of patients with obesity was significantly low (0.8%) in the study population, which is most likely due to poor documentation, and the actual prevalence may be higher than this. Data obtained from other studies showed that high body mass index is associated with dyslipidemia. In Pakistan, data showed that about 38.6% were obese, 34.7% were overweight, 25.7% were standard, and 1% were underweight. Among the obese patients, dyslipidemia was present in 34.5% of patients. Dyslipidemia was prevalent in overweight patients [26]. Our study population may also have a higher prevalence of obesity; hence, proper documentation is essential to address this.

In patients with a very high risk of developing ASCVD, such as those with ACS, it is recommended to achieve a lipid reduction of the target LDL-C goal of less than 1.4 mmol/L or more than 50% from baseline [11]. Data from this study revealed that 52% of patients admitted with ACS were not screened for LDL-C levels within 48 hours of admission. This triggered a problematic strategy for the baseline LDL-C goal as recommended by the guideline [35,38].

Furthermore, this study revealed that 113 (90.6%) patients did not undergo subsequent LDL-C follow-up after three to six months of initiating statin therapy. Among the 11 (9.4%) follow-ups, there were still hiccups in the serial documentation of LDL-C results. The majority (82%) of patients from this study did not achieve the guideline-recommended target LDL-C levels on statin monotherapy even though 94% of patients were covered and maintained on high-intensity statin, as evidenced in different other studies such as Pravastatin or Atorvastatin Evaluation and Infection Therapy-Thrombolysis in Myocardial Infarction 22 and A-TO-Z trials into decreasing cardiovascular mortality compared to the standard dose of statin [39-41]. Consistent with other studies, this study revealed that a minority of patients were able to attain a guideline-recommended target level of LDL-C of less than 1.4 mmol/L or more than 50% reduction in LDL-C from baseline despite being exposed to high-intensity statin with documented good adherence. Similar findings were observed in a nationwide register in the United States, revealing that one out of three patients with ACS had not attained the target LDL-C levels [37]. Other studies revealed nearly equal levels of lipid-lowering effect on the LDL-C, with a 20% reduction in major adverse cardiovascular events. This variation in clinical outcomes is also attributed to the pleomorphic effect of different statins, which may occur differently. High-intensity statin use has beneficially shown promising outcomes in the risk of recurrent angina and revascularization [42]. None of the patients in this study were on nonstatin therapy despite the robust evidence on the significant lowering of LDL-C and its role in improving cardiovascular mortality [34,35]. However, studies have not established how low the LDL-C levels may need to be set based on each case, below which further reduction will not yield any cardiovascular benefit [15].

Nevertheless, a meta-analysis involving eight statin trials revealed that more than 40% of the patients on high-dose statins did not achieve an LDL-C less than 1.4 mmol/L. This result was attributed to factors including interindividual variability [18]. This meta-analysis showed that some individuals achieved the guideline-recommended LDL-C levels while others responded poorly, making the management of lipids suboptimal. Some factors that cause variability in response to lipid-lowering with statins include age, sex, weight, diet, and physical activity [43]. Variable responses were also observed due to nonadherence to statins for various reasons, including adverse drug effects and depression [44]. Some studies have revealed a genetic predisposition to nonresponse to statins, whereby the 3-hydroxy 3-methylglutaryl coenzyme A reductase gene (HMGCR) was shown to have been associated with lower efficacy of pravastatin treatment [45,46]. In the Treating to New Target trial, apolipoprotein E (APOE), PCSK9, and HMGCR variants were also associated with statin efficacy, including atorvastatin [47]. Furthermore, the Justification for the Use of Statins in Primary Prevention: an Intervention Trial Evaluating Rosuvastatin trial observed that variants of ABCG2, lipoprotein a, APOE, and PCSK9 were involved in response to rosuvastatin in different individuals [48]. This study also agrees that there could be interindividual variability in response to statin therapy.

The key limitations of this study include its retrospective nature and a smaller sample size. Another limitation is that we did not include patients who were admitted for routine coronary angioplasty, known to have ischemic heart disease. This could have resulted in patients with severe CAD being excluded from this study. Furthermore, this is a single-center study, and results may not be generalizable to other centers. Future multicenter studies involving a higher number of patients are recommended to confirm these findings and to recommend changes in clinical practice to improve compliance with guidelines.

## Conclusions

This study revealed that half of the patients with ACS admitted at the JKCI had LDL-C screening within the guideline recommended 48 hours after presentation to the hospital. The study revealed good practice in initiating and maintaining high-intensity statins in these patients with ACS as secondary prevention. Nevertheless, the LDL-C control within the ESC guideline-recommended dose is still suboptimal, data that were consistent with other studies. Further studies assessing the issues around the low uptake of nonstatin therapies in patients with ACS would be useful. The low uptake of these therapies could be due to the limited availability of these drugs, which is due to the high cost and difficulty of local availability. Individual variability also triggers various responses to statin therapy among different individuals. None of the patients in this study had a nonstatin therapy. More emphasis should be placed on the prompt screening of LDL-C within 48 hours of hospital presentation. The follow-up LDL-C levels after four to six weeks and six months since the initiation of statin therapy are also essential to evaluate the response to statin and optimize therapy to achieve optimal LDL-C control. Further larger size and multicentered studies are recommended to evaluate compliance with current national and international guidelines and to improve patients care.
